# Transcription profiling of fertilization and early seed development events in a solanaceous species using a 7.7 K cDNA microarray from *Solanum chacoense *ovules

**DOI:** 10.1186/1471-2229-10-174

**Published:** 2010-08-12

**Authors:** Faiza Tebbji, André Nantel, Daniel P Matton

**Affiliations:** 1 Institut de recherche en biologie végétale, Département de sciences biologiques, Université de Montréal, 4101 rue Sherbrooke est, Montréal, Québec, H1X 2B2, Canada; 2 Biotechnology Research Institute, National Research Council, 6100 Royalmount Avenue, Montreal, QC, H4P 2R2, Canada

## Abstract

**Background:**

To provide a broad analysis of gene expression changes in developing embryos from a solanaceous species, we produced amplicon-derived microarrays with 7741 ESTs isolated from *Solanum chacoense *ovules bearing embryos from all developmental stages. Our aims were to: 1) identify genes expressed in a tissue-specific and temporal-specific manner; 2) define clusters of genes showing similar patterns of spatial and temporal expression; and 3) identify stage-specific or transition-specific candidate genes for further functional genomic analyses.

**Results:**

We analyzed gene expression during *S. chacoense *embryogenesis in a series of experiments with probes derived from ovules isolated before and after fertilization (from 0 to 22 days after pollination), and from leaves, anthers, and styles. From the 6374 unigenes present in our array, 1024 genes were differentially expressed (≥ ± 2 fold change, p value ≤ 0.01) in fertilized ovules compared to unfertilized ovules and only limited expression overlap was observed between these genes and the genes expressed in the other tissues tested, with the vast majority of the fertilization-regulated genes specifically or predominantly expressed in ovules (955 genes). During embryogenesis three major expression profiles corresponding to early, middle and late stages of embryo development were identified. From the early and middle stages, a large number of genes corresponding to cell cycle, DNA processing, signal transduction, and transcriptional regulation were found. Defense and stress response-related genes were found in all stages of embryo development. Protein biosynthesis genes, genes coding for ribosomal proteins and other components of the translation machinery were highly expressed in embryos during the early stage. Genes for protein degradation were overrepresented later in the middle and late stages of embryo development. As expected, storage protein transcripts accumulated predominantly in the late stage of embryo development.

**Conclusion:**

Our analysis provides the first study in a solanaceous species of the transcriptional program that takes place during the early phases of plant reproductive development, including all embryogenesis steps during a comprehensive time-course. Our comparative expression profiling strategy between fertilized and unfertilized ovules identified a subset of genes specifically or predominantly expressed in ovules while a closer analysis between each consecutive time point allowed the identification of a subset of stage-specific and transition-specific genes.

## Background

Angiosperm sexual reproduction starts when pollen is transferred from the anther to the stigma. After hydration and germination, the pollen tube carrying two sperm cells enters the embryo sac through the micropyle of the ovule and penetrates one of the synergid where it discharges its contents. One sperm cell fuses with the egg cell and the resultant zygote develops into the embryo. The central cell unites with the second sperm cell to form a triploid primary endosperm cell that develops into the endosperm [1-5]. Thus, the double fertilization event initiates the development of two interconnected multicellular structures, the diploid embryo and the triploid endosperm. Embryogenesis patterning starts with an asymmetric cell division that produces a small apical cell, that ultimately becomes the embryo, and a large basal cell (leading to the suspensor) that functions to provide nutrients from the endosperm to the growing embryo [6]. Embryogenesis in higher plants can be divided conceptually into three overlapping phases [7-9]. The first phase involves morphogenesis and pattern formation, during which the polar axis of the plant body is defined by specification of the shoot and root apices, and the embryonic tissue and organ systems are formed (pattern formation/cell proliferation/cell division). During the second phase (maturation), storage reserves start to accumulate in the maturing embryo, and, in the third phase, the embryo prepares for desiccation and enters a period of developmental arrest. Successful embryogenesis thus leads to seed development. In *Arabidopsis *embryogenesis is rapid, requiring only 14 days after pollination to produce the desiccated mature seed [10]. In solanaceous species, embryogenesis spans a much longer period. For example, in *Solanum phureja*, embryo maturation, excluding desiccation, is only completed 27 days after pollination [11].

Classification of gene expression patterns associated with specific stages of embryo and seed development and functional understanding of the encoded genes are critical for understanding the molecular and biochemical events associated with embryogenesis. Most of the knowledge concerning the genetic program activated during embryogenesis of higher plants comes from the study of mutants. Several mutants affecting embryonic development were identified in numerous plant species including *A. thaliana*, maize, rice, and tomato through various mutagenesis strategies [12-26]. These analyses enabled the identification and characterization of several genes that play key roles in plant embryonic development. However, mutagenesis strategies alone cannot identify all of the genes that are potentially involved in a biological process.

In the past few years, several differential screening techniques (including differential display, subtracted libraries, differential hybridization, etc.) have made it possible to characterize genes differentially expressed during embryogenesis [27,28]. More recently, other methods such as transcriptional profiling have allowed us to visualize global changes in transcript abundance in a spatial, temporal or conditional way. Genome-wide transcription profiling is an important and powerful tool leading to the generation of testable hypotheses for novel processes not yet characterized at the molecular level. Its usefulness has been demonstrated in the investigation of transcriptional programs occurring in a variety of developmental processes such as fruit ripening, seed development, flower development, embryo development, defense response to pathogens, and the response to wounding [29-44]. Now that the technology has matured, microarray data are routinely correlated with other methods that measure RNA expression levels [45,46].

Recently we completed a medium scale EST sequencing project from normalized cDNA libraries made from *Solanum chacoense *ovules in order to identify signal transduction components involved in fertilization and early seed development [47]. *Solanum chacoense*, a diploid (2n = 2x = 24) and self-incompatible close relative of the potato and tomato, produces a large numbers of easily isolated ovules inside a fleshy tomato-like fruit. It also bears tubers, albeit small, like all the members of the *Solanum *Petota section, and is also considered an important source of interesting genetic traits for its high resistance to more than 20 pests and diseases [48]. Flowers of *S. chacoense *are actinomorphic with a 5-lobed calyx and corolla, five stamens, and a 2-carpellate superior ovary. *S. chacoense *display homomorphic self-incompatibility of the gametophytic type involving S-RNases. The presence of a gametophytic self-incompatibility barrier was used to dissect the molecular events occurring at precise time points following pollination and fertilization since it enables synchronization of fertilization. These EST were used to construct cDNA microarrays consisting of 7,741 cDNAs spotted in duplicate, which were used to investigate the regulation of gene expression during fertilization and early seed development events from 0 to 22 days after pollination (DAP). Our aims were to: 1) identify genes expressed in a tissue-specific and temporal-specific manner; 2) define clusters of genes showing similar patterns of spatial and temporal expression; and 3) identify stage-specific or transition-specific candidate genes for further functional analyses.

## Methods

### *Solanum chacoense *cDNA clones and inserts amplification

We have previously used a subtraction screen on two cDNA libraries covering embryo development from early fertilization events (6-12 hours post-fertilization) to ovules bearing late torpedo stage embryos as a method to produce an EST pool enriched for weakly expressed messenger RNAs. From roughly 50,000 colonies spotted on nylon membranes, 8000 colonies that displayed a hybridization signal corresponding to the lowest 20% were selected for further analyses. 7741 good sequences were obtained and these comprised 6374 unigenes [47]. Inserts of these cDNA clones were PCR amplified using flanking primers complementary to vector sequences upstream and downstream of the cDNA inserts. Plasmid templates (1-2 ng) were added to 100 μl PCR mixture containing 0.2 mM of each nucleotide, 1 μM of each primer, 1.5 mM of MgCl_2 _and 10 units of Taq DNA Polymerase. Inserts were amplified for 36 cycles (95°C for 30 sec, 54°C for 30 sec, 72°C for 2 min), with an initial denaturation at 95°C for 2 min and final extension at 72°C for 6 min. Two (2) μl of each reaction were separated on 1.5% agarose gels to confirm amplification quantity and quality. PCR products were purified using MultiScreen^® ^PCR_96 _filter plates (Millipore, Billerica, MA, USA), and lyophilized. In general, the amount of each PCR product was greater than 5 μg and the average insert size was around 1000 bp.

### cDNA microarray preparation

PCR products were resuspended in 10 μl of 50% DMSO and arrayed from 384 well microtiter plates onto UltraGAPS™ slides (Corning incorporated, Corning, NY, USA). A total of 7741 ESTs spotted in duplicate along with a variety of controls, including buffer-only spots and a dilution series of a plasmid harboring the *Candida albicans Ece1 *gene.

### Plant materials and RNA isolation

The diploid (2n = 2x = 24) and self-incompatible wild potato species *Solanum chacoense *Bitt. was greenhouse grown with an average photoperiod of 14-16 h per day. The genotypes used were originally obtained from the USDA Agricultural Research Service, NRSP-6 Potato Genebank (Potato Introduction Station, Sturgeon Bay, WI, USA). Plant material was collected from female progenitor, *S. chacoense *genotype G4 (S_12 _and S_14 _self-incompatibility alleles) [49]. For fertilization-related events, the fully compatible *S. chacoense *genotype V22 (S_11 _and S_13 _self-incompatibility alleles) was used as pollen donor. Plants were hand pollinated and ovules were collected between 0 and 22 days after pollination (DAP) and used for RNA extraction and probe preparations. Leaf, style and anther tissues were collected from plant genotype G4 grown under the same conditions as above. All samples for RNA preparation were quick-frozen in liquid nitrogen and ground to a powder with a mortar and pestle. Total RNA was extracted using the TRIzol^® ^Reagent according to the manufacturer's protocol (Invitrogen, Burlington, ON, Canada). The yield and purity of RNA were assessed by determination of absorbance at both 260 nm and 280 nm. RNA was only used when the ratio Abs_260 __nm_/Abs_280 nm _was higher than 1.7. RNA integrity was checked by both agarose gel 1% and with the RNA 6000 Nano Assay Kit and the Agilent 2100 Bioanalyzer. RNA from unfertilized ovules served as control.

### Design of microarray experiments

To monitor the expression pattern from genes involved in fertilization and embryogenesis processes, flowers were hand-pollinated and ovules were isolated every two days during a 22 days period after pollination. Four independent biological replicates were produced from each time points. In addition, to isolate genes specifically or predominantly expressed in ovules, four biological replicates of leaf, anther, and style tissue mRNA preparations were individually hybridized against unfertilized ovule mRNAs and compared with the data obtained from unfertilized and fertilized ovules at various time points after pollination. To estimate reproducibility and to produce control data for statistical analysis, a large number of unfertilized ovules were isolated and separated between seven independent control groups. RNA from randomly selected pairs of control was hybridized on six microarrays.

### Preparation of fluorescent probes

Thirty (30) μg of total RNA, 1.5 μl oligo (dT)_21 _(100 pmol/μl), and water to a volume of 36 μl was denatured at 65°C for 10 min and cooled to room temperature for 5 min. The reverse transcription reaction was performed at 42°C for 2 h after addition of 3 μl dNTP-minus dCTP (6.67 mM each), 1 μl dCTP (2 mM), 4 μl DTT (100 mM), 8 μl 5× first strand buffer (Invitrogen), 2 μl of cyanine 3-dCTP (1 mM) or cyanine 5-dCTP (1 mM; Perkin Elmer-Cetus/NEN, Boston, MA, catalog # NEL999) and 2 μl of SuperScript II (200 units/μl, Invitrogen). After incubation at 42°C, 0.05 units each of RNase A and RNase H (Invitrogen) were added and the reaction mix was incubated at 37°C for 30 min. The probes were then purified using CyScribe GFX Purification Kit (GE Healthcare Bio-Sciences Inc., Baie d'Urfé, QC, Canada) according to the manufacturer's instructions. Incorporation efficiency of the CyDye probes was measured using the ND-1000 Nano drop spectrophotometer (NanoDrop, Wilmington, DE, USA). In general, optimal amounts are 20 pmol of dye incorporated with an incorporation efficiency of between 20-50 labeled nucleotides per 1000 nucleotides. Probes were then air-dried.

### Hybridization

Slides were prehybridized at 42°C for at least 1 hour, with 50 μl of a solution containing 5× SSC, 0.1% SDS and 1% BSA. The two cDNA targets were pooled together, and mixed with the hybridization buffer to a volume of 50 μl Dig Ease Hybridization buffer (Roche Applied Science, Mississauga, ON, Canada), 2.5 μl tRNA (Roche Applied Science) and 2.5 μl of Sonicated Salmon Sperm DNA (10 mg/ml; Invitrogen). The hybridization solution was heat denatured at 95°C for 3 min, cooled to room temperature, and applied onto the DNA microarray slides for overnight hybridization at 42°C. To account for the possibility of dye bias, half of the hybridizations were performed in the Cy3/Cy5 configuration, and half in the Cy5/Cy3 configuration. The microarray slides were covered with a 24 × 60-mm glass coverslip (Fisher Scientific, Ottawa, ON, Canada). During all hybridization steps the hybridization chamber was kept at high humidity level. Immediately before hybridization, the DNA microarray slides were washed twice with 0.1× SSC at room temperature for 5 min and once with water for 30 sec and centrifuged at 800 rpm for 3 min. The DNA microarray slides were kept dry for a minimal amount of time before hybridization. Afterward, slides were completely immersed in a large volume of washing buffer, and the coverslips were carefully removed before washing twice for 10 min at 42°C with 1× SSC, 0.1% SDS, twice for 10 min at 37°C with 0.1× SSC, 0.1% SDS, and finally, with quick consecutive washes in three 0.1× SSC baths. DNA chips were air dried and stored protected from light until scanning.

### Microarray scanning and data analysis

The DNA microarray slides were scanned with a ScanArray Lite microarray scanner (Perkin Elmer-Cetus, Wellesley, CA) at 10-μm/pixel resolution. The fluorescence intensities were quantified with QuantArray software (Perkin Elmer-Cetus; versions 2.0 and 3.0). Microarray data normalization and analysis was performed in GeneSpring GX software version 7.3 (Agilent Technologies, Santa Clara, CA, USA). Raw intensities were normalized with a Lowess curve using 20% of the data to fit each point. To identify transcripts with a significant change in abundance, the fluorescence ratios from each time point were compared to the fluorescence ratios from 6 control hybridizations using the Welch t-test and the Benjamini and Hochberg False Discovery Rate. We selected genes with a p-value ≤ 0.01 and further restricted the lists to transcripts whose change in abundance was greater than 2-fold. The use of such a stringent gene selection method that uses both multiple testing correction and a fold-change cutoff did not significantly affect our conclusions. GeneSpring GX was also used for hierarchical clustering and to detect significant overlaps between gene lists. Functional categorization was performed according to the Gene Ontology (GO) Consortium and Arabidopsis consortium information (http://www.geneontology.org/). Translated *S. chacoense *sequences were sorted into 15 functional categories by sequence comparison. The data discussed in this publication have been deposited in NCBI's Gene Expression Omnibus [50] and are accessible through GEO Series accession number GSE21552 (http://www.ncbi.nlm.nih.gov/geo/query/acc.cgi?acc=GSE21552).

### Real-time PCR

cDNA samples were synthesized from 2 μg of 2 independent preparations of total RNA using the SuperScript^® ^II Reverse Transcriptase (Invitrogen). The RNA was denatured at 70°C for 10 min. The total volume was adjusted to 40 μl by adding 8 μl of 5× RT buffer, 2 pmoles of oligo dT 25 primer, 4 μl of 0.1 mM DTT, 2 μl of 10 mM dNTP, 200 units of SuperScript^® ^II Reverse Transcriptase and 19 μl of water. The mixture was then incubated 1 hour at 42°C. The resulting first strand cDNA was used for real-time PCR amplification experiments. Ovule RNAs from three different developmental stages (4, 12, and 22 days after pollination) were compared. RNAs from unfertilized ovules were used as the calibration tissue. Duplicate quantitative assays for each tissue were performed with the SYBR Green Master mix (Invitrogen) according to the manufacturer's instructions. For real-time PCR amplification, the following PCR program was used: 50°C for 2 min, 95°C for 10 min, 95°C for 15 s, 60°C for 1 min; steps 3 and 4 were repeated 40 times in Mx4000^® ^Multiplex QPCR System (Stratagene, La Jolla, CA, USA). The relative quantification analysis was performed using the comparative ΔΔCt method [51]. To evaluate the gene expression level, the results were normalized using an ubiquitin gene (DN977330) as control. Primer sequences used in real time quantitative PCR are summarized in Additional file 1.

### Tissue fixation and optical microscopy observation

Ovules were fixed in FAA for 24 h at 4°C (50% ethanol, 1.35% formaldehyde, and 5% glacial acetic acid). Samples were then dehydrated in an increasing series of ethanol baths (from 30% to pure ethanol). Microscopic observations were taken on an AxioImager M1 microscope equipped with an Axio Cam MRm camera (Carl Zeiss Canada, Toronto, ON, Canada).

## Results

### Microarray spotting and evaluation

We have previously generated expressed sequence tags (ESTs) derived from fertilized ovule cDNA libraries covering embryo development from zygote to late torpedo stages in *Solanum chacoense *Bitt [47]. The 7741 ESTs corresponded to weakly expressed mRNAs obtained through a subtraction selection screen that produced a highly enriched unigene set (6374 unigenes) corresponding to 82% of the total ESTs sequenced. To provide a glimpse into the ovule transcriptome during embryogenesis in a solanaceous species and to identify a subset of ovule-specific and stage-specific genes comprising this transcriptome, these ESTs were used to construct amplicon-based cDNA microarrays. In the present study, we used a comparative expression profiling strategy to analyze gene expression profiles in ovules from 0 to 22 days after pollination.

For microarray production, the ESTs were amplified by PCR with vector-specific primers. The PCR products were purified, verified for single clone amplification, normalized, and arrayed onto aminosilane-coated glass surfaces. To increase the reliability of the detected signals, each PCR sample was spotted twice resulting in 15,482 data points for each array. Several controls were also spotted to verify the reliability of our hybridization. After each spotting runs, samples of printed arrays were visualized with a fluorescent dye to control evenness of DNA deposition and spot morphology.

**Figure 1:**
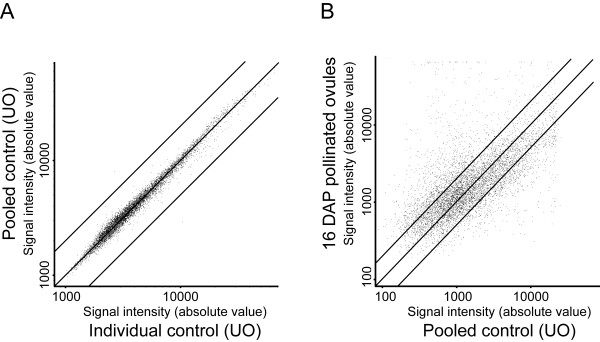
**Gene expression profiling of unfertilized and fertilized ovule samples**. Scatter plots comparing the average fluorescence intensities in microarray experiments measuring the change in transcript abundance between A, pooled control and individual control; or B, 16 DAP pollinated ovules versus unfertilized ovules. The individual control samples were different from the pooled one that is used as the universal control in all our experiments. Black transversal lines show the ±2 fold change.

Sixty-two (62) hybridizations corresponding to 15 experiments were performed (see material and methods for details). Twelve experiments consisted of the comparison of ovule tissues harvested at 2, 4, 6, 8, 10, 12, 14, 16, 18, 20 and 22 days after pollination (DAP) with unfertilized ovules (control, 0 DAP). To determine the percentage of genes specifically or predominantly expressed in ovules, three experiments that compared RNA from unfertilized ovules to leaf, style, and anther tissues were also conducted. Due to the large amount of control tissue material needed for each time point comparison and for each replicate (unfertilized ovules corresponding to 0 DAP), the control RNA obtained were pooled from different replicates and used for comparison with the various time points and other tissues. Pooling RNA before labeling has the advantage of reducing the variation due to biological replication and sample handling [52]. To estimate this variation we also competitively hybridized cDNA probes from six additional control samples collected from unfertilized ovule against the pooled control. The six individual control samples were different from the pooled one that was used as the universal control in all our experiments. The correlation coefficient between the normalized intensities of the two channels was of 0.98, and only few spots showed occasional differences in intensities above 2-fold (Figure 1a). This very high correlation coefficient gives a strong indication that biological replication is not a significant source of variation. For each experiment, four biological replicates were collected from the same greenhouse and from a large number of plants. For each individual replicate, all the necessary material was collected at once. Each hybridization was performed against the pooled control RNA sample (unfertilized ovules, UO). To account for the possibility of dye bias, half of the hybridizations were performed in the Cy3/Cy5 configuration and half in the Cy5/Cy3 configuration.

### Expression profiling of genes involved in fertilization and embryogenesis

To monitor gene expression profiles during fertilization and early embryogenesis, total RNA samples from UO to 22 DAP were isolated, labeled, and hybridized to the 7.7 K microarray. A fraction of the ovules from each sample was set aside and fixed. These ovules were cleared and analyzed by differential interference contrast (DIC) microscopy to ensure that homogenous developmental stages were used in the various developmental time points used. Strict pollination timing was accomplished through manual pollinations with pollen from a fully compatible *S. chacoense *genotype on anthesis day. Fertilization occurs between 36 and 42 hours following pollination in *S. chacoense *[53]. Since *S. chacoense *is a self-incompatible species this prevents self-fertilization and eliminates the need for emasculation, which could induce a bias in gene expression through a wounding response. Furthermore, since anther dehiscence is not completed at the time of anthesis while the pistil is already fully receptive, this reduces the risk of self-pollen landing on the stigma that could trigger a self-incompatible response.

**Figure 2:**
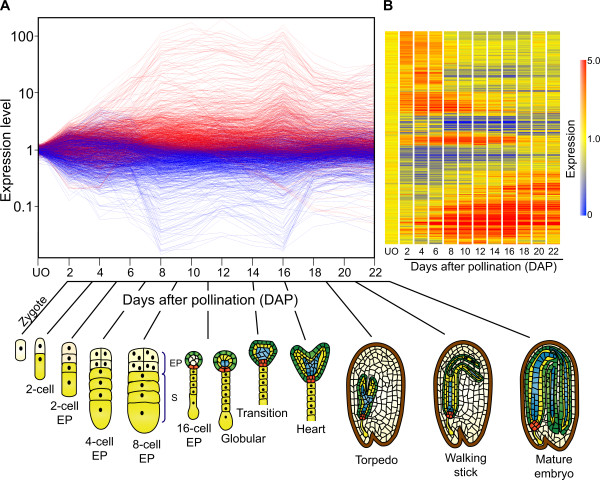
**Transcriptional changes in *S. chacoense *ovules across an embryo developmental time course**. A, Each line represents a probe that was spotted on the microarray while each time points of embryo developmental stage are represented in X-axis. The Y-axis shows the log of the normalized fluorescence ratios. Indicated in red are the genes expressed to levels higher than the median value. Genes indicated in blue are expressed to lower levels than the median value. The corresponding embryo developmental stages for each time point was ascertain by ovule clearings and observed by DIC microscopy and represented with a schematic figure. B, Cluster analysis using unsupervised hierarchical clustering of 1024 genes that exhibit a statistically significant change between all samples (*P *< 0.01). The analysis was performed using condition tree clustering on all samples. Each row represents a different gene, and each column displays gene expressions at each time point (0 to 22 DAP). Each experimental data point is colored according to the change in fluorescence ratio at that time point: data values displayed as red and blue represent increased and reduced expression, respectively while data values in yellow are not differentially expressed when compared to unfertilized ovules.

For each time point, four biological replicates were used, with half labeled as Cy3 cDNA probes and the other half as Cy5 cDNA probes. Using Anova testing, along with a Benjamini and Hochberg multiple testing correction algorithm, 1997 transcripts showed a statistically significant change in abundance (p ≤ 0.01) in at least one of the time points compared to the control hybridizations (Figure 2A). Amongst those, 1024 showed a greater than ±2-fold variation in expression in at least one of the time points (Additional file 2). These differentially expressed genes were grouped according to the similarity of their expression profiles using two dimensional hierarchical clustering (Figure 2B) [54]. These results clearly segregate the dataset amongst three major groups of genes that specify early (494 genes), middle (604 genes), and late (203 genes) stages of embryogenesis (Figure 2A and 2B). Embryonic stages as determined by ovule clearing and microscopical observation encompassing each time point are schematically represented (Figure 2A). From 0 to 6 DAP (early stage), embryo development corresponded to the post fertilization stages that include embryos from the zygotic stage to 8-celled embryo proper. From 8 to 16 DAP (middle stage) ovules mainly bore embryos from the 16 cell to heart stages, and from 18 to 22 DAP (late stage) embryo developmental stages encompassed torpedo, walking stick, and mature embryos.

Figure 2 presents the global profiling during all stages of embryogenesis. In order to better illustrate the dynamic and magnitude of these changes in gene expression, and the distribution of the signal intensities obtained from each probe, an example of a scatter plot representing the data from a single time point comparing pollinated ovule (16 DAP) versus unfertilized ovule is shown in Figure 1B. This representation shows the global distribution of all the genes spotted on the microarray slide at 16 DAP vs. UO. The scatter plot showed a positive correlation between fertilized ovules at 16 DAP vs. unfertilized ovule for most of the genes, with the majority of the genes analyzed showing a lesser than ±2 fold difference in expression when compared to UO across the full intensity range. Genes that show a greater than ±2 fold expression difference (735 genes) are also evenly distributed across all intensity range up to 150 fold change with a group of genes that show signal saturation. Comparing each time point scatter plots against the UO experiment (Figure 1) clearly showed that, for each time point, a group of genes are significantly modulated. All time point scatter plot comparisons gave similar distributions (data not shown).

### Analysis of genes specifically or predominantly expressed in ovule tissues

**Figure 3:**
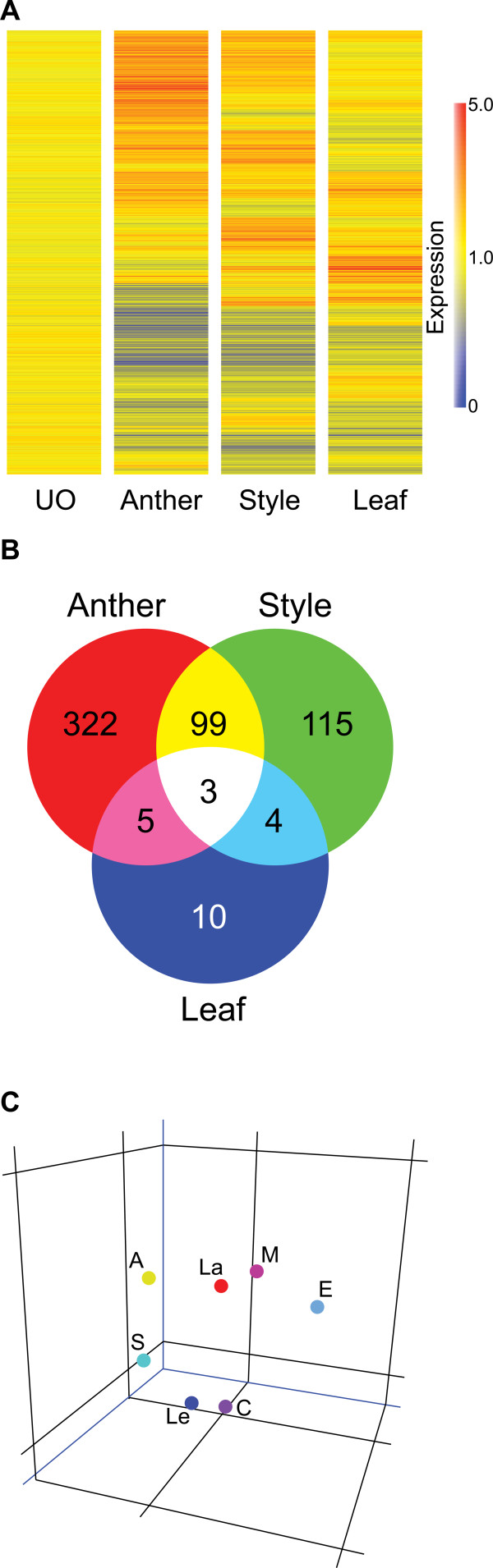
**Tissue-specific comparison between ovule, style, anther and leaf tissues**. A, Cluster analysis of the transcriptional profiles obtained from leaves, styles, and anthers. The clustering was performed using condition tree clustering on all samples. Each row represents a different gene, and each column displays gene expressions at different tissues (UO, style, leaf and anther). Data values displayed as red and blue represent elevated and reduced expression, respectively. A total of 601 genes with a statistically significant change in transcript abundance of ≥2-fold in at least one tissue compared to unfertilized ovules (p value < 0.01) are found. B, Venn diagram showing overlap between lists of of genes differentially expressed in anther, style and leaf tissues. C, Principal Components Analysis (PCA) of various tissue types (A: Anthers; S: Styles; Le: Leaves; C: unfertilized ovules. E, M, L: early, middle and late stages of embryo development, respectively) based on the the expression profile of 1376 genes including 558 that are differentienlly expressed in at least one tissue from styles, leaves and anthers and 1024 transcripts that are regulated in ovule which resulted in a clear differentiation distributed between 7 classifications.

To determine the percentage of ovule-specific genes in our EST pool, RNA samples from leaf, anther, and style tissues of *S. chacoense *were compared to the UO pool sample. Each microarray experiment was performed four times using, in all cases, independently isolated RNA samples as starting material (four biological replicates). Statistical analyses showed that 558 genes (out of all ESTs) showed a significant variation in transcript abundance (≥ ± 2-fold coupled with p values ≤ 0.01) in at least one tissue (leaf, style, or anther) when compared to UO (Figure 3A). A Venn diagram analysis of modulated genes from leaves, anthers, and styles shows that, from the 558 genes, 3 genes were differentially expressed in all tissues compared to ovules; 322 genes are differentially expressed only in anthers; 10 genes are differentially expressed only in leaves; and 115 genes are differentially expressed only in styles. Four (4) genes are co-regulated in leaves and styles; 5 genes are co-regulated in leaves and anthers; and 99 genes are co-regulated in anthers and styles (Figure 3B).

From these 558 genes, 262 genes were up-regulated (≥2-fold variation) and 296 genes were down-regulated (≤2-fold change) when compared to UO (Additional file 3). We thus considered that the transcripts corresponding to these 296 genes were predominantly expressed in UO. From the 262 transcripts that were predominantly expressed in styles, anthers, or leaves, 69 genes were also differentially expressed in ovules after pollination but their induction level was less than the one observed in the other tissues tested (<2-fold). A principal components analysis of these genes clearly indicates that the subset of genes expressed in non-ovule tissues is quite different than those expressed in ovules (Figure 3C). Thus, these results confirm that a large number of the ESTs on our microarrays can be considered ovule-specific or ovule-predominant. Out of the 1024 genes that showed more than ±2-fold variation in expression after fertilization, 955 (1024 - 69, 93.2%) are considered specifically or predominantly expressed in ovule tissues.

Amongst these genes, a small group (1.15%) was modulated by more than 100-fold during embryogenesis, 2.1% of the genes were modulated by ±50 to 100-fold, 4.8% between ±20 and 50-fold, and 6.1% between ±10 and 20-fold (Additional file 4). The most highly expressed genes comprised almost exclusively genes involved in proteolysis and peak transcript accumulation occurred between 8 and 16 DAP (Figure 2A). Genes involved in proteolytic function were also highly represented in the ≥50 to 100-fold and ≥20 to 50-fold up-regulated genes and covered various classes of proteases including serine-type peptidases (carboxypeptidases, subtilases or subtilisin-like serine protease), aspartic-type peptidases, and cysteine-type peptidases. Apart from genes involved in proteolysis, other highy up-regulated genes (≥10-100 fold) included numerous lipid transfer proteins (LTP) and non-specific lipid transfer proteins (nsLTP) that are involved in various biological processes including plant defense, pollen tube adhesion and guidance, embryo patterning and cell wall biogenesis [55-60], as well as proteinase inhibitors that are involved in plant defense responses and early seed development [61,62].

Amongst the genes found to be strongly repressed in the following combined categories (from ≤10 to 20, ≤20 to 50, ≤50 to 100-fold), most corresponded to genes classified as being involved in stress responses with a high representation of metallocarboxypeptidase inhibitor (MCPI) genes. One such MCPI was characterized by Martineau et al. (1991) as being highly expressed in anthesis stage ovaries in tomato (*Solanum lycopersicum*) while decreasing quite rapidly during fruit development, with an estimated 10-fold drop [63,64]. The three most similar *S. chacoense *orthologs to the tomato MCPI were also found in the 10 to 20-fold down-regulaetd genes (Additional file 4).

### Functional classification of differentially expressed genes during fertilization and early seed development

**Figure 4:**
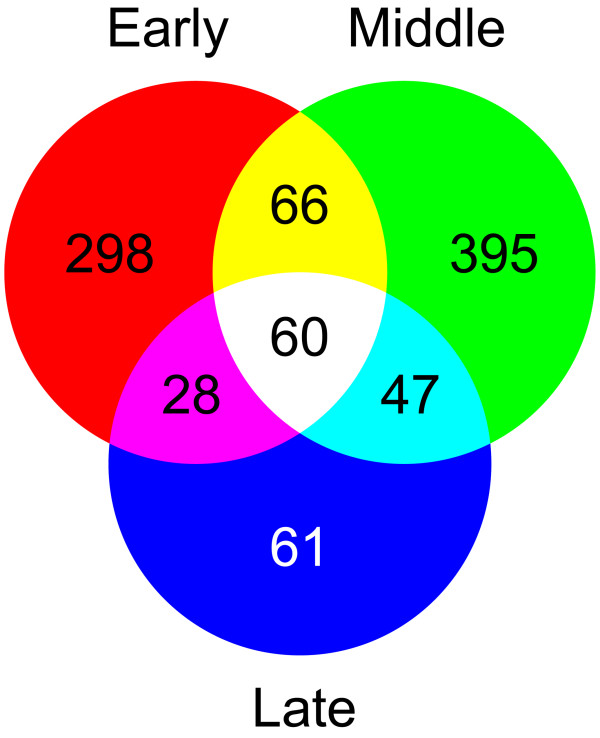
**Venn diagram showing overlap between gene lists that show significant changes in transcript abundance during the three major embryo development stages (early, middle and late stages)**.

The hierarchical gene clustering presented in Figure 2B showed that, following fertilization, the ovule-expressed gene pool could be clearly divided into three major groups that specified early, middle, and late stages of embryogenesis. A Venn diagram analysis of the genes predominantly expressed in fertilized ovules indicates that 298 genes are differentially expressed during the early developmental stage, 395 genes only during the middle stage, and 61 genes only during the late stage (Figure 4). Sixty-six (66) genes are differentially expressed in early and middle stages, 28 genes in early and late stages and 47 genes in middle and late stages. Sixty (60) genes are differentially expressed in all embryo development stages (Additional file 5). Since the ESTs used to build the microarray were isolated from ovules harvested at various time following pollination, they encompass all different developmental stages of the embryo. The microarray analysis was thus sensitive enough to separate them between the major developmental transitions. This also suggests a highly specific expression program for each separate stage, with little overlap between the three major stages defined.

**Figure 5:**
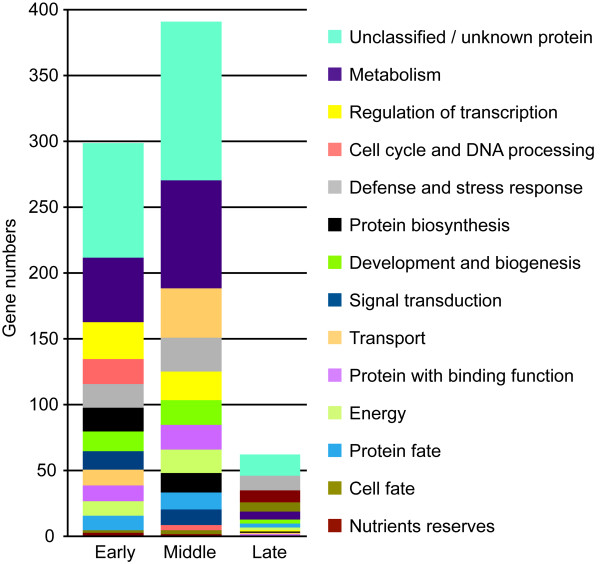
**Functional annotations of the 955 genes (passing the significance filter: *P *< 0.01 and 2 fold cut-off) during the three major embryo developmental stages**. The color keys show the designated gene class annotations.

To determine whether the differentially expressed genes were involved in similar biological processes, transcript profiling data was correlated with the Gene Ontology classification based on both the *S. chacoense *sequences and their closest orthologs in *Arabidopsis thaliana*. The functional categories of these genes are given in Figure 5. As expected, the largest category in every stage consisted of genes with unknown function or genes with no significant homology/no hit (29.8%), as observed in fully sequenced genomes [65]. For the early and middle stages, the largest functional categories included metabolism (49 and 83 genes respectively), regulation of transcription (28 and 22 genes respectively), energy (11 and 18 transcripts respectively), signal transduction (14 and 12 transcripts respectively), protein fate (11 and 13 transcripts respectively) and proteins with binding functions (12 and 19 transcripts respectively).

One functional category of genes that was highly overrepresented in the early stage compared to the middle and late stages corresponded to genes related to the cell cycle & DNA processing category (19 in early stage, with 18 transcripts up-regulated, and 4 in middle stage with 3 genes up-regulated), probably reflecting the high cell division activity observed in the endosperm that precedes the development of the embryo. In solanaceous species, embryo division starts when the endosperm has reached 24-48 cells [11]. For the protein biosynthesis category, 18 genes were differentially expressed in early stage with a large proportion of up-regulated genes (14 transcripts). An inverse situation was found in the middle stage, where only 3 transcripts were up-regulated out of 15 genes for this functional category. Interestingly, for the protein fate group, 3 genes out of the 11 found were up-regulated in the early stage, while 9 genes out of 13 were up-regulated in the middle stage, suggesting a coordinate inverse regulation of protein biosynthesis and fate during these two stages. For the following gene categories corresponding to development and biogenesis (15 in early and 19 in middle), and transport (12 in early and 38 in middle), the number of transcribed genes was higher in the middle stage when compared to the early stage.

Genes associated with nutrient reserves tend to specify the late stage, as expected. The defense and stress responses category was important in all three stages. Differentially expressed genes associated with the signal transduction and regulation of transcription classes were almost nonexistent in the late stage, comprising only 0.13% (1 out of 754 genes) of the transcripts while they comprised 5.57% (42 out of 754 genes) and 6.36% (48 out of 754 genes) in early and middle stages respectively.

### Isolation of stage-specific genes

**Figure 6:**
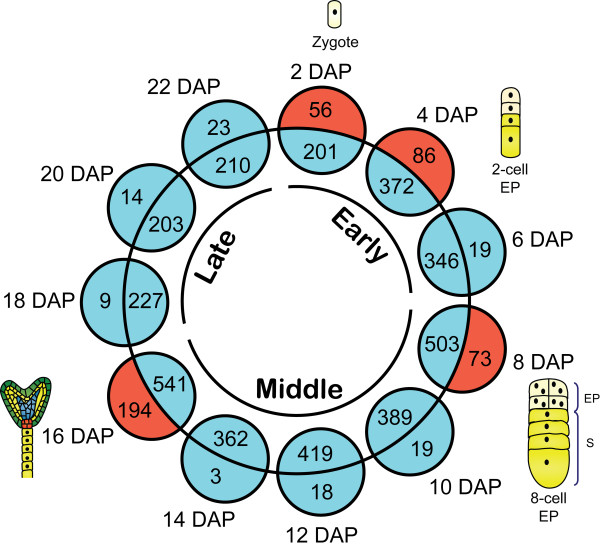
**Graphical representation of the number of stage-specific genes identified at each time point of embryo development (Volcano plot using Student's t-test analysis taking p value < 0.01 and 2 fold cut off)**. To find specific genes in each time, a Venn diagram was used between each time point and the total of genes remaining in the other stages. Numbers in the inner circle are genes shared by at least another time point. Numbers in the outer circle are genes considered stage-specific by the Volcano analysis.

To characterize in more detail the transcriptional changes taking place between developmental stages, a different analysis was undertaken for each time point during embryogenesis from 0 to 22 DAP. A Volcano Plot with Student's t-test analysis was used to identify genes that were specifically expressed at only one time point when compared to UO (2 DAP vs UO, 4 DAP vs UO, etc) during embryogenesis, with a p value ≤ 0.01 and a ≥ ± 2 fold cut-off. Depending on the time point chosen, from 217 to 735 genes were significantly modulated at any single time point and between 3 and 194 genes were specifically modulated during a single time point (Figure 6). Thus, these represent candidates for stage-specific or stage-predominant expressed genes. Of particular interest, four time points (2, 4, 8 and 16 DAP) had a high proportion (from 1/8 to 1/4) of their genes specifically modulated only at the observed time point. In the early zygotic phase at 2 DAP, 56 of 257 modulated genes (22%) were only expressed at this stage. At 4 DAP, when ovules bear embryos with a 2-cell embryo proper, 86 of 458 modulated genes (19%) were stage-specific. The last two time points enriched in stage-specific genes are found at the beginning and the end of the middle phase at 8 and 16 DAP, respectively. At 8 DAP, when ovules bear mostly embryos undergoing transition from a 4-cell to an 8-cell embryo proper 73 of 576 genes modulated genes (13%) were stage-specific while at 16 DAP, when ovules bear mostly heart-stage embryos, 194 of the 735 modulated genes (26%) were stage-specific. Thus, although the majority of the genes were differentially expressed during several embryo stages, genes differentially expressed only at a single time point were also found for all stages in variable numbers. Additional file 6 provides the list of these stage-specific genes. At 2 DAP, half the stage-specific modulated genes were classified as part of the functional category covering translation. At 4 DAP, we noted the accumulation of the stress and defense related genes including heat shock proteins (12.34%) and genes coding for proteins with binding function (9.87%). The major functional category represented at 8 DAP was related to energy (12.12%). A specific group of cell cycle regulation (3.03%) and chromosome organization and biogenesis (6.06%) were also found. Finally, the 16 DAP time point was specially enriched in three gene ontology (GO) categories: hormone related genes (4.84%), signal transduction (6.06%) and transport (6.06%).

### Functional classification of differentially expressed genes in style, anther and leaf

**Figure 7:**
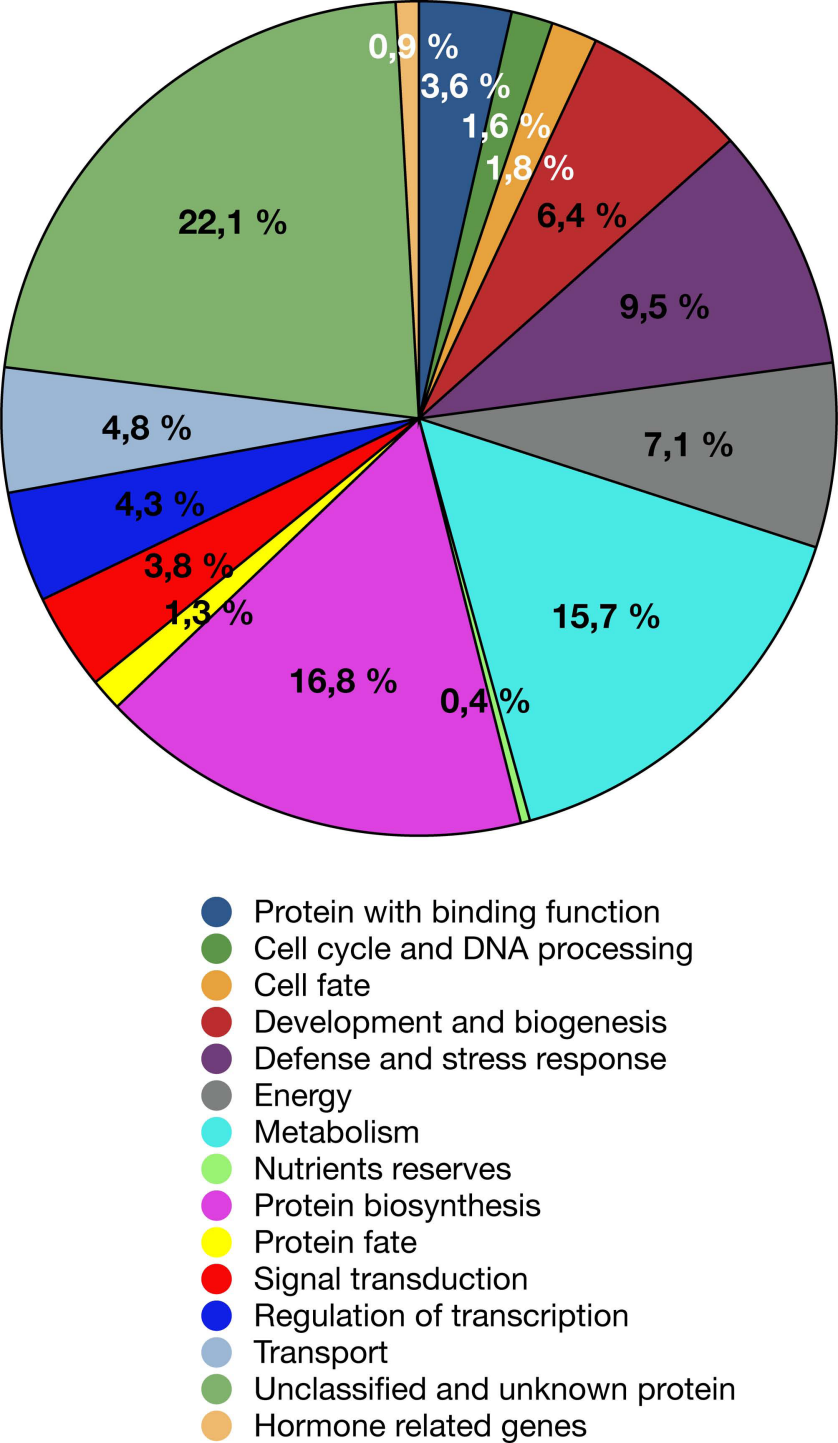
**Distribution of 558 differentially expressed (by 2 fold-change, *P *< 0.01) genes in anthers styles and leaves in various biological process categories**. The color key shows the designated gene class annotations.

The 558 genes that were differentially expressed in other tissues when compared to UO were grouped into different functional categories (Additional file 3) according to their predicted gene products, based on the Gene Ontology (GO) Consortium through *S. chacoense *sequences and their closest orthologs in *Arabidopsis *(Figure 7). Apart from a large class of unknown and unclassified proteins (22.14%), the largest functional groups observed were protein synthesis (16.78%), metabolism (15.71%), energy (7.14%), stress and defense related genes (9.46%), development and biogenesis (6.42%), transport (4.82%), regulation of transcription (4.28%), signal transduction (3.75%), proteins with binding function (3.57%), cell cycle and DNA processing (1.6%), and protein fate (1.25%).

Comparison of the categories found in these tissues and in ovules showed a completely different enrichment in functional categories (compare Figures 5 and 7). Whereas globally in ovule tissues (all stages taken together), we observed a large proportion of genes related to signal transduction and transcription, a very active cell cycle, and an up-regulation of genes associated with protein synthesis, in the other tissues tested these categories were less represented and even in the most represented category, protein biosynthesis, almost all genes were down-regulated (92 out of 94 genes).

### Validation of microarray data by real-time PCR

**Figure 8:**
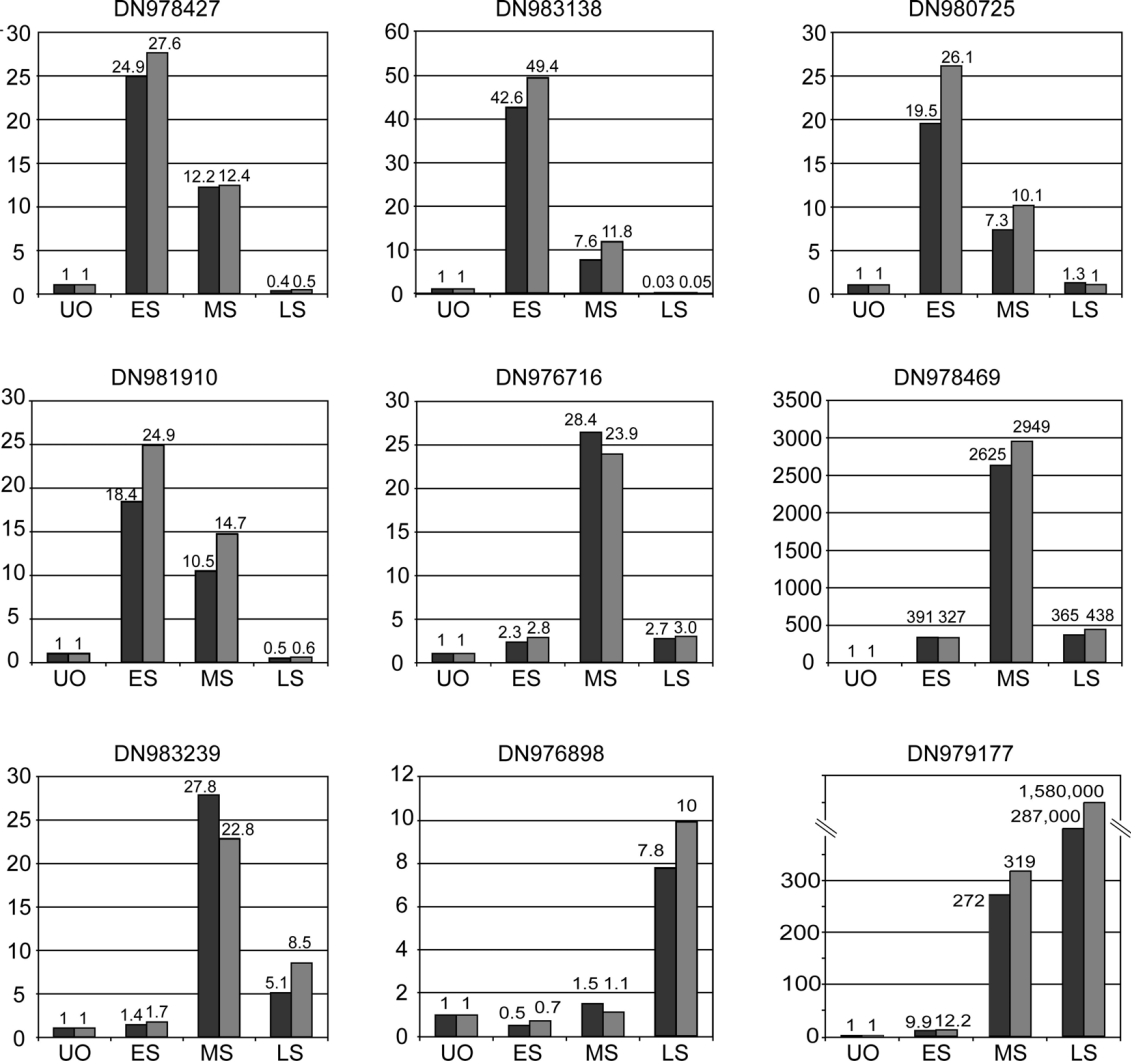
**Quantitative real-time PCR (qRT-PCR) of selected genes representing the early, middle and late stage of embryo development**. qRT-PCR was performed with two independent samples at each point.

In order to confirm the validity of differentially expressed genes identified by cDNA microarray analysis, we performed real time RT-PCR experiments on candidates from the three major groups of genes that specify early, middle, and late stages of embryogenesis. Quantitative RT-PCR analyses were performed on RNA extracted from ovules four days after pollination corresponding to the early stage of embryo development; from ovules 12 days after pollination corresponding to the middle stage; and from ovules 22 days after pollination corresponding to the late stage. Unfertilized ovules were used as the calibration tissue and the ubiquitin amplicon as the internal control to normalize the data. Quantitative RT-PCR confirmed the expression pattern for all the nine genes chosen from the microarray analysis: DN980725, DN981910, DN978427, and DN983138 that specify the early stage of the embryo development; DN976716, DN983239, and DN978469 which specify the middle stage; DN979177 and DN976898 which specify the late stage (Figure 8). In all cases, the quantitative RT-PCR validation indicated that differential expression detected by microarray experiment was highly reliable.

## Discussion

Until recently, most methodologies used to study the molecular mechanism involved in plant embryogenesis were based on mutagenesis approaches, and were mainly conducted in a few plant species, namely, *Arabidopsis*, rice and maize. These have allowed the characterization of numerous and informative mutants affecting early embryogenesis [19,21,22,66-73]. In recent years, candidate gene studies involving gene-profiling technologies have enabled the global visualization of spatial and temporal differential gene expression patterns, thus being complementary to mutagenesis approaches while also being able to characterize new key genes inaccessible through mutant screens. Only few such studies targeting plant embryogenesis have been conducted [36-38,42,43]. In this study, we have analyzed for the first time a large transcriptomic dataset of temporal gene expression in ovules from a solanaceous species, *S. chacoense*, by comparing the gene expression profiles of fertilized ovules versus unfertilized ovules.

Apart from slower embryo development, two other major differences between the model plant *A. thaliana *and *S. chacoense *should be considered. Both refer to early embryogenesis stages. Contrary to *A. thaliana*, which follows the Onagrad type, *S. chacoense *follows the Solanad type of early zygote divisions. In both cases, the first division of the zygote is transverse and asymmetrical, giving rise to two cells of different developmental fates. The smaller apical cell gives rise to the embryo proper, while the larger basal cell develops into the suspensor. In *A. thaliana *the first two divisions of the apical cell are longitudinal, while they are transversal in *S. chacoense *[74]. Endosperm also differs in whether nuclear divisions are followed by cellular divisions. Three variants are commonly recognized and classified as cellular, nuclear or helobial [75]. In cellular endosperms, as found in *S. chacoense*, cell-wall formation follows the first division of the primary endosperm nucleus (PEN). In helobial endosperms, wall formation follows the first PEN division, producing two chambers, which vary in subsequent cellularization. In nuclear endosperms, as found in *A. thaliana*, walls do not develop between the free nuclei.

We have covered the whole embryo development process from zygote to mature embryo and have identified 955 genes (≥ ± 2 fold change, p ≤ 0.01) that are specifically or predominantly expressed in ovules compared to their expression profile in leaves, styles or anthers. Many differentially expressed genes encode proteins with putative regulatory functions, and most of them have not yet been characterized. Overall, in ovule tissues, a large proportion of genes related to transport, signal transduction and regulation of transcription were modulated, and an up-regulation of genes associated with cell cycle and protein synthesis was noticed. In the other tissues tested (style, anthers and leaves), these categories were less represented and, even in the most represented category, protein biosynthesis, almost all genes were down-regulated (92 from 94 genes). The data obtained indicates that some gene categories are overrepresented in some tissues or organs. Interestingly, we found that expression of MADS-box, MYB, AP2-EREBP and YABBY transcription factor families were overrepresented in the transcriptional regulation groups during the early and middle stages of embryo development as well as in anthers tissues, but absent in the leaves and style. This suggests that these genes might play an important role in defining structural and functional identity of reproductive tissues. Consistent with this, many transcription factors from the above-mentioned gene families have been previously shown to play major roles during plant reproduction [36,76] and floral organ development [77-81]. Therefore, genes identified as being preferentially expressed in a given tissue probably have an important function in this tissue [26,82-85].

### Specific functional category enrichment in the three major embryonic developmental stages

**Figure 9:**
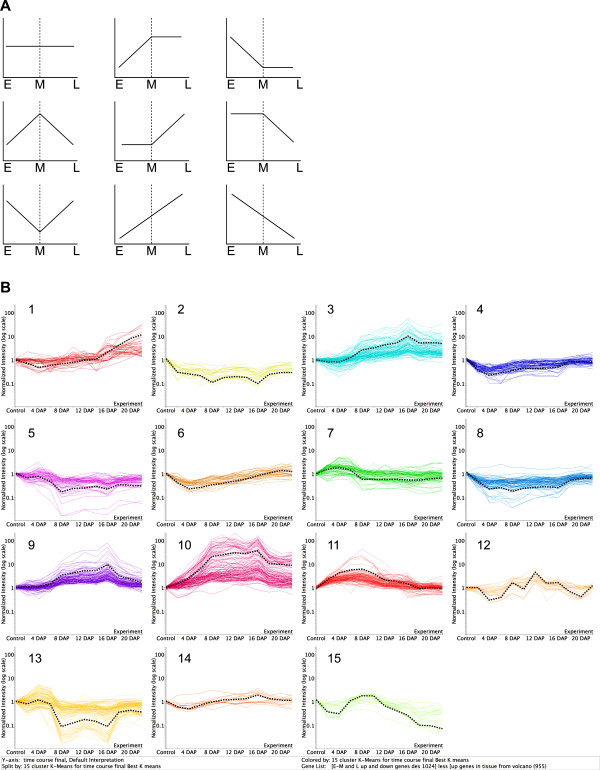
**K-means clustering using Pearson Correlation (GeneSpring version 7.2) during an embryo development time course**. A, Models of dynamic gene expression patterns representing all the nine different patterns found based on the three point time (early, middle and late). B, Clustering of specific differentially expressed genes in the ovules based on 15 clusters representing all the stages of embryonic development from 0 to 22 DAP. The black discontinues line represent the average of gene profile.

Differentially expressed genes in ovules were grouped according to the similarity of their expression profiles using unsupervised hierarchical clustering algorithms. Clustering results segregated the dataset amongst three major groups of genes that specified early (from zygote to 16-cell stage), middle (16-cell to heart stage) and late (heart to mature embryo) stages of embryogenesis (Figure 2). This result indicates a clear distinction in transcriptionnal profiles between early, middle and late stages of embryogenesis, as determined with the principal components analysis (Figure 3), the little overlap between the modulated genes (Figure 4), as well as the specific enrichment in the corresponding functional categories (Figure 5). K-means clustering using Pearson correlation was also used to separate the expression patterns during the embryo developmental time course. Considering that three major expression profiles are found, representing early, middle and late stages of embryonic development, a maximum of nine patterns can be expected (Figure 9a). Hennig et al. (2004) also proposed nine models of dynamic expression patterns for genes involved in reproductive development in *Arabidopsis *based on three different stages: before, during and after pollination [36]. Spencer et al. (2007) found that seven distinct expression patterns were present within the apical and basal section of the embryo, during the globular, heart, and torpedo stage embryos [37]. The two missing profiles are most probably the consequence of the absence of earlier stages, before the globular stage. When considering less global trends, as depicted in Figure 6 for stage-specific genes, a higher number of specific and slightly different patterns can be obtained, based on the 12 individual time points. Figure 9b depicts 15 such patterns that can be derived from the analysis of our time-course every second day from 0 to 22 DAP and that include the nine dynamic expression patterns representing early, middle and late stages of embryonic development. Although the majority of functional groups are shared between several stages, some of these clusters show specific functional categories enrichment (Additional file 6). The first cluster (cluster 1) includes genes involved in protein storage and specifies the late stages of embryogenesis. Cluster 7 corresponds to the early stage (2 and 4 DAP) and is enriched in genes classified in the protein biosynthesis category. Cluster 11 shows a peak at 8 DAP and is enriched in genes of the nucleosome assembly and cell cycle regulation group. Clusters 3 and 9 show a profile with a pronounced peak at 16 DAP, corresponding to the transition from heart to torpedo stages. These two clusters are enriched in functional categories corresponding to proteins with binding function, lipid metabolism, transport and genes implicated in development and biogenesis. Cluster 10 represents the transition of the early stage to middle (8 DAP) and middle to the late (16 DAP) and is characterized by a large number of genes involved in proteolysis, lipid metabolism and transport, development and protein folding.

Based on the global trend from the three major clusters specifying early, middle and late stages of embryogenesis, we found that the transcriptional pattern of some functional categories could also specify embryo developmental stages. These included metabolism, transcriptional regulation, cell cycle, protein biosynthesis, defense and stress response, development and cell growth, signal transduction, transport, proteins with binding function, protein fate and energy, which are represented in early and/or the middle stage. The late embryonic stage was characterized, in addition to some goups found in the early and middle stages, by the cell fate and nutrient reserves functional categories (Figure 5). A comparison with the closest study published by Spencer and collaborators (2007) in *Arabidopsis*, shows a larger representation of functional groups in our dataset. Spencer and collegues found 1872 and 1226 differentially expressed genes in the apical and basal time course, respectively, using a p value of 0.05 for genes whose expression was significantly different from a value of 1. They then chose the 200 most highly expressed genes (100 in apical and 100 in basal tissues) between the developmental stages studied. In our dataset, we used different criteria, a p value of 0.01 and cut off of ≥ ± 2 fold in each time point compared to the same control. Our GO groups were assigned to all the genes that were specific and differentially expressed in the early (zygote to 16 cell), middle (globular to heart) and late (torpedo to mature embryo) stages of embryogenesis. While in the *Arabidopsis *published data [37], the transition from globular to heart stage is characterized by the up regulation of genes in functional groups corresponding to energy production, metabolism, protein biosynthesis, signal transduction and transcription, in our dataset, the middle stage, which include this transition, is enriched in the same functional groups except for the protein biosynthesis category which characterized the early embryonic stage (from zygote to almost the globular stage). In addition, other enriched functional categories were up-regulated in the middle stage including protein with binding function, development and cell growth, protein fate and transport. The late stage was characterized by the up-regulation of the nutrient reserve category. It was reported that the storage proteins typically accumulate only from the mid-maturation phase onwards, when cell division is completed and the basic form of the embryo has developed [10]. Given that the plant body pattern is already established and that the seedling continues to mature, it was expected to have similar transcriptional profile from torpedo to mature embryo and have an up regulation of nutrient reserves at all these late stages, which is indeed what we observed. Thus, during embryo development, some functional group characterized specific stages while other groups are shared between different embryogenesis stages. Indeed, the up regulation of genes related to cell cycle and protein biosynthesis characterized the early stage; protein with binding function, protein fate, cell-cell communication and transcription specified the middle stage; and reserve proteins were more abundant in late stage. Energy and transport functional groups were represented in the early and middle stages while stress and defense related genes were highly represented during all developmental stages.

Protein biosynthesis, including ribosomal proteins (RP) and components of the translation machinery, were up-regulated in the early embryonic stage but then down-regulated in the middle stage. Spencer and colleagues [37] found a similar trend but it appears to be delayed compared to our data as it shows a significant up-regulation of genes involved in protein synthesis between the heart stage and the torpedo stage in their apical embryo time course, and between the globular and the heart stage in their basal embryo time course. These embryo developmental stages would be included in our middle stage, where we observed a decrease in protein synthesis components when compared to our early stage. This could be representative of the transition from early embryogenesis to the maturation and protein accumulation phase typical of the later embryogenesis stages [10]. Conversely, protein fate genes were down-regulated in the early stage but up-regulated in the middle stage. Genes belonging to the cell cycle category were exclusively up-regulated in the early stage. Up-regulation of the protein biosynthesis category specified the second and fourth day after pollination, afterward, these genes were either down-regulated or showed no differential expression during the later time points. This finding reflects the need to produce sufficient amounts of these essential proteins and the indispensability of protein synthesis in the actively developing seeds. Conversely, genes involved in protein degradation were overrepresented later in middle and late stages. Spencer et al. (2007), found the same enrichement order, while in the basal time course, from the globular to the heart stage, they showed the up regulation of protein biosynthesis, then the protein fate group was overrepresented from the heart to the torpedo stage and the protein biosynthesis group was absent. The over-expression of these particular categories of genes illustrates the high energetic requirements of embryo tissue and suggests that other regulatory mechanisms at the post-transcriptional and posttranslational levels are important during the embryogenesis. Consistent with this, other interesting genes were found including genes coding for ubiquitin, and proteinase inibitors. Indeed, transcriptional and post-transcriptional control have been described for the proteinase inhibitors of soybean [86] and other legume seed proteins [87]; and ubiquitination was already found to be an important post-translational regulatory process of the unfertilized egg cell in wheat [88].

Examination of the cell cycle and DNA processing categories revealed a prevalence of gene up-regulation from 2 to 16 DAP. B-type cyclin-dependant kinases (CDKs) involved in the control of the G_2_/M progression in plants [89] as well as a large number of S-phase markers such as Histone 1, 2, 3 and 4, involved in DNA replication and modification [90-92] were significantly enriched and up-regulated. This is not unexpected since cell division in the embryo starts after fertilization and continues until the heart stage [8]. It has also been shown that protein synthesis and the cell cycle are associated at two levels [93]. On the one hand, protein synthesis is required for entry and progression through the cell cycle; on the other hand, protein biosynthesis is itself modified during cell division steps. This finding is in line with our data set. In the early stage, the up-regulation of RPs, translation and elongation factors coincide with the overexpression of histones. Markedly, in the middle stage, proteolysis genes represented by a large number of ubiquitin/proteasome-related genes, proteases, peptidases, and subtilases, were activated, suggesting active degradation of proteins by proteolysis, while protein biosynthesis genes remain expressed at a basal, non-modulated level. Importance of ubiquitine/proteasome-related genes during embryogenesis has also been reported to strongly affect the progression of the *Arabidopsis *embryo from the globular stage onward [94]. Altogether, this suggests important post-translational protein regulation in cell cycle progression during embryogenesis.

The set of differentially expressed genes modulated during the early and middle phases also contained many signal transduction components and transcriptional regulators. Among them were 24 signaling proteins and 50 transcriptional regulators. In the signal transduction category, one calmodulin, most similar to the *Arabidopsis *CaM7 [95], and one calmodulin-like gene, most similar to the *AtCML24 *[96] were isolated. Interestingly, three *Arabidopsis *SIP4 orthologs (SOS3 kinase-interacting protein 4 or CBL-interacting protein kinase 11 (CIPK11), also characterized in tobacco as ACRE216 - Avr9/Cf-9 rapidly elicited protein 216) [97,98] and an ortholog of the *Arabidopsis *CBL-interacting protein kinase 5 (CIPK5) were also isolated, suggesting an important role for protein phosphorylation and calcium signaling during sexual reproductive development. Of the protein kinases modulated during embryogenesis, only one coded for a receptor-like kinase, *ScORK11 *[47]. Interestingly, from the EST pool that was used to make the cDNA microarray, we had previously isolated 30 different RLKs with 28 being predominantly expressed in ovary tissues or young developing fruits, and 23 being transcriptionaly induced following fertilization [47]. This discrepancy highlights the difference in sensitivity of the methodologies used, e.g. microarray analysis (this study) vs. quantitative RT-PCR analysis [47]. A shaggy-like kinase gene similar to the *Arabidopsis ATSK41 *[99], an IRE-Like AGC kinase gene [100] and an ortholog of the *Arabidopsis *RAB GTPase homolog B1C [101] were also isolated. Most of these have been previously shown to be involved in various stress responses and none had been shown to play a role in embryogenesis. Considering the high number of differentially expressed genes (100/955) classified as involved in defense and/or stress response during all stages of embryogenesis, this also emphasize their potential roles in response to pollination and during reproductive development.

Several genes implicated in embryonic patterning were found in the first two major stages. Amongst these, PROTODERMAL FACTOR 1 (*PDF1*) and TOPLESS-RELATED 1 (*TPR1*) are up-regulated in the early and in middle stage, respectively. PDF1 is a predicted cell wall protein restricted to the protoderm of the embryo following tangential divisions between the 8 and 16-cell stages [102] and specifies the first element of the radial pattern [12]. Since the *S. chacoense PDF1 *ortholog shares the same expression pattern as the *Arabidopsis PDF1 *with an expression peak at the 16-cell stage (2.24-fold at 8-cell and 3-fold at 16-cell stages) this protein most probably exerts the same function in solanaceous species. The TPR1 gene is involved in maintenance of shoot fates during *A. thaliana *embryogenesis defining the apical pole of embryo [103]. Transcriptional activation of this protein in our study suggests that the *S. chacoense *TPR1 ortholog is developmentally regulated during embryogenesis and might also act as a determinant of embryo polarity in solanaceous species.

Many members of the MADS box family are expressed at higher levels during embryogenesis [104]. Interestingly, a protein identified as an ortholog of the *A. thaliana AGL11/SEEDSTICK *gene was up-regulated during the middle stage. *AGL11/STK *is required for normal development of the funiculus, an umbilical-cord-like structure that connects the developing seed to the placenta, and for dispersal of the seeds when the fruit matures [105,106]. Furthermore, *AGL11/SEEDSTICK *is also expressed during embryogenesis in ovules as determined from publicly available microarray data (BAR, http://www.bar.utoronto.ca) with a high activity in stage 3 seeds (globular stage of embryo development). Then its expression decreases during the middle stage and increases again in the late stage of seed development. Rounsley et al. also showed that *AGL11/SEEDSTICK *expression is also maintained after pollination through late seed development [76]. MADS-box genes are known to act as homeotic selector genes determining floral organ identity and also as floral meristem identity genes [77,78,80,81]. Our profiling study highlights the potential role of MADS-box transcription factors during the early stages of reproductive development. Several others studies showed an important differential expression of transcription factors, including MADS-box, from various families during pollination, fertilization and seed development [36-40,107-109].

### Biotic and abiotic stress-responsive genes

More than 100 genes out of the 955 differentially expressed genes are classified as potentially involved in defense and/or stress response during all stages. This suggests that these defense-related genes might also function in response to pollination and during embryo development, in addition to their contribution in defense against pathogens [110,111]. Among activated genes in our dataset, those corresponding to proteins of the subtilase family were induced by two- to 137-fold. An ortholog of the ARA12 Subtilisin-like serine protease showed a two- to 73-fold change in *S. chacoense*. Golldack and colleagues showed by *in situ *hybridization that the ARA12 subtilisin-like serine protease was present at higher level in pistils, ovules and anthers, but also responded to stress and pathogen stimuli [112]. Several other pathogenesis-related proteins were also up-regulated after pollination during embryo development, including proteinase inhibitors, Thaumatin-like protein, PR10, β-1,3-glucanase-like protein and elicitor inducible chitinases. A strong increase in transcript levels, from 2- to 118-fold, of non-specific lipid transfer proteins (nsLTPs) has been found during the middle and late stages. These proteins are member of a superfamily of seed protein called prolamins that, in addition to storage, plays important roles in plant defense responses [113,114]. The nsLTPs have been reported in a number of plants, including solanaceous plants [115], and are involved in lipid transfer and in the formation of a protective covering of cutin and suberin layers over plant surfaces. Proteomics analysis of embryo and endosperm from germinating tomato seeds revealed a high level of nsLTPs in the tomato endosperm suggesting a role in the mobilization of lipids from the endosperm to the embryo and probably in defense against infection during germination [116]. Recently, Vriezen et al (2009) also reported 54 stress or defense-related genes that were expressed in tomato ovary tissues prior to fertilization with the vast majority (47/54) being down-regulated after fertilization. In our case, in 2 DAP ovaries compared to unfertilized ovaries, 54 stress or defense-response genes were regulated, with 34 down-regulated genes. Furthermore, when looking at the whole profile from 2 to 22 DAP, a much more dynamic and variable profile for these genes is observed, precluding any global generalization over the whole time-course. The activation of defense-related genes during embryogenesis is probably due to the fact that the plant protects embryo development by increasing the activation of defense and stress-related genes. Alternatively, some of these stress-related genes might exert other functions as for the POP2 gene that is also involved in pollen tube adhesion and growth [117,118] and in oxidative stress response [119], and for the HSP70-1 chaperone, involved in development and abiotic stress responses in *Arabidopsis *[120]. Altogether, this suggests an overlap between fertilization/embryogenesis and stress and defense response pathways. This was also reported by Lan et al. (2005) in rice, revealing a cross talk in genetic programs controlling pollination/fertilization and stress responses [121].

### Identification of stage or transition specific candidate genes during embryo development

To identify stage-specific or transition-specific candidate genes we used a Volcano Plot analysis to identify genes that were specifically expressed at only one time point during embryogenesis with a p value ≤ 0.01 and a ≥ ± 2 fold cut off. As mentioned before, 4 time points (2, 4, 8 and 16 DAP) had a high proportion of stage-specific or stage-predominant candidates genes. At 16 DAP, when ovules bear mostly heart-stage embryos, 26% of modulated genes were stage-specific and were enriched in up-regulated genes from the signal transduction, transport, and hormone-related genes gene ontology (GO) categories, when compared to the GO profiles of the other time points. Of the genes orthologous to the *Arabidopsis *EMB genes required for normal embryo development [22], six (*EMB 2171 *- 2 DAP, *EMB 1738 *- 4 DAP, *EMB 2473 *- 4 DAP, *EMB 1473 *- 8 DAP, *EMB 1345 *- 16 DAP, and *EMB 1990 *- 16 DAP) were found to be stage-specific in the Volcano analysis. *EMB 2171 *and *EMB 1473 *code for the mitochondrial targeted 60S L17 RP and the chloroplastic 50S L13 RP, respectively. *Arabidopsis *mutant plants corresponding to these genes display embryos arrested at the globular stage (http://www.seedgenes.org/index.html). These two genes had been previsouly showed to be highly expressed during early embryo development in *S. chacoense *[122]. *EMB 1738 *codes for the *CYP51G1 *obtusifoliol 14-α demethelylase gene, a gene involved in lipid signaling [123]. In *emb 1738 *plants, two-thirds of the embryos are arrested at the early transition to heart stage [22]. *EMB 2473/MIRO1 *codes for a GTPase required for embryogenesis that also affects pollen mitochondria morphology [124]. *MIRO1 *mutants display embryos arrested at the zygotic to the 4-cell stage. *EMB 1345 *codes for a WD40 protein similar to the CIA1 protein involved in iron/sulfur protein biogenesis [125], that interacts with the Willm's tumor suppressor protein WT1 [126]. In *emb 1345 *mutants, only 3% of the analyzed plants displayed globular embryos while the remaining plants had no detectable embryos (http://www.seedgenes.org/index.html). The *EMB 1990 *gene codes for an chloroplastic putative integral membrane protein [127]. In *emb 1990 *mutants, two-thirds of the embryos are arrested at the globular stage while 13% have a cotyledon terminal phenotype (http://www.seedgenes.org/index.html).

Two families of auxin-regulated genes (the *Aux/IAA*, and *GH3 *families) have been identified among the transcripts that were specifically found in 16 DAP heart-stage embryos. Auxins are important signaling molecules involved in many developmental processes in plants. Identification of embryo-defective mutants affected in the transport or the auxin signaling pathway indicates that auxins play various roles in coordinating the organization of the embryo by providing positional information [128]. During embryogenesis, it is particularly important in the specification of the apical cell after the first zygotic division [129], the formation of the root meristem from the hypophysis [130-132], and the establishment of bilateral symmetry [133]. Apical-to-basal transport of the hormone auxin is initiated at the early globular stage and plays a key role in regulation of many aspects of embryonic pattern formation in plant [134,135]. In dicots, auxin is first found in the endosperm and only later is it detectable in the embryo itself with a special distribution starting with a maximal accumulation in the apical cell until the 32-cell-embryo stage. At later stages an inverse pattern of auxin maxima is observed [129,136,137]. Amongst the auxin-regulated genes found in our transcriptomic analysis, we noticed the up-regulation of the *GH3 *gene, *YDK1 *(*YADOKARI *1). Takase et al (2004), found a dwarf mutant, named *ya do Kari 1-D *(*ydk1-D*), which had a T-DNA insertion proximal this *GH3 *gene. The *ydk1-D *mutant is dominant and has a short hypocotyl, a short primary root, reduced lateral root number, and reduced apical dominance, suggesting that *YDK1 *may function as a negative component in auxin signaling by regulating auxin activity [138]. A second gene of interest is *IAA13*, a member of the auxin response factors family which is required for the axialization of the embryo [128,139-143]. Weijers et al, showed that stabilization of BDL/IAA12 or its sister protein IAA13, prevents MP/ARF5-dependent embryonic root formation. A dominant mutation that renders IAA13 insensitive to auxin-dependent degradation results in elimination of embryonic axis: the cell at the base of the proembryo divides abnormally and never elongates or become organized in files. The seedlings produce a short peg in place of the hypocotyl and root and also show fused cotyledons with reduced vasculature. *IAA13 *is initially expressed in the apical daughter of the zygote and in all cells of the proembryo, but it becomes restricted to the provascular cells by the midglobular stage [128]. Although the shoot apical meristem (SAM) gradually develops during embryogenesis, it appears as a distinct histological entity somehow late during embryogenesis [144], around the time where we observed peak expression of numerous auxin-related genes.

## Conclusion

Our analysis provides the first study, to our knowledge in a solanaceous species, of the transcriptional program that takes place during the early phases of plant reproductive development, including all embryogenesis steps. We used a comparative expression profiling strategy between fertilized and unfertilized ovules to provide an insight into the embryo transcriptome in *S. chacoense *and to identify a subset of genes comprising this transcriptome. Several potential regulators of fertilization and early seed development have been identified. We identified 1024 genes (955 genes were specifically expressed in ovules tissues when compared to others tissues) that were specifically regulated during these developmental stages, along with many highly stage-specific genes. Although the biological function of most genes remains to be determined, the identification of genes involved in reproductive processes in solanaceous species that produces embryos much more slowly than model species like *A. thaliana*, will enable the selection of stage-specific genes that will deepen our understanding of complex stage transition processes as well as pinpoint potential targets for improving yield and seed quality by conventional breeding or biotechnological approaches.

## Authors' contributions

FT carried out the experiments, interpreted the data, and drafted the manuscript. AN gave extensive advices on microarray data analysis and edited the paper. DPM conceived the research, directed the overall project, and played a major part in writing and results interpretation. All authors have read and approved the final manuscript.

## Supplementary Material

Additional file 1 **Quantitative PCR primers**. Primer sequences used in the real time quantitative PCR expression analysis of selected genes representing candidates from the early, middle, and late stages of embryogenesis shown in figure 8. Click here for file

Additional file 2 **Differential expression of 1024 genes amongst during embryogenesis**. Using Anova testing along with a Benjamini and Hochberg multiple testing correction algorithm, 1024 transcripts showed a statistically significant change in abundance (p value ≤ 0.01) and ±2-fold variation in expression in at least one of the time points during embryo development compared to the unfertilized ovule control. Click here for file

Additional file 3 **Differential expression of genes in anthers, style, and leaf tissues compared to unfertilized ovules**. RNA samples from leaf, anther, and style tissues of *S. chacoense *were compared to the unfertilized ovules (UO) pool sample. Each microarray experiment was performed in quadruplicate using, in all cases, independently isolated RNA samples as starting material (four biological replicates). A total of 558 genes (262 genes were up-regulated and 296 genes were down-regulated) showed a significant variation in transcript abundance (≥ ± 2-fold coupled with a p value ≤ 0.01) in at least one tissue (leaf, style, or anther) when compared to UO. Click here for file

Additional file 4 **Differential expression of genes during embryo development showing a greater than ±10-fold change**. Using Anova testing, along with a Benjamini and Hochberg multiple testing correction algorithm, 135 transcripts showed a statistically significant change in abundance (p value ≤ 0.01) and ±10-fold variation in expression in at least one time point during embryo development compared to the unfertilized ovule control. Click here for file

Additional file 5 **Differential expression of genes amongst the three major stages (early, middle, and late) of embryogenesis**. Using Anova testing along with a Benjamini and Hochberg multiple testing correction algorithm, 1024 genes were differentially expressed (≥ ± 2 fold change, p value ≤ 0.01) in fertilized ovules compared to unfertilized ovules. Only limited expression overlap was observed between these genes and the genes expressed in the other tissues tested, with the vast majority of the fertilization-regulated genes (955) being specifically or predominantly expressed in ovules. The hierarchical gene clustering analysis showed that, following fertilization, the ovule-expressed gene pool could be clearly divided into three major groups that specified early, middle, and late stages of embryogenesis. A Venn diagram analysis of the genes predominantly expressed in fertilized ovules indicated that 298 genes are differentially expressed during the early developmental stage, 395 genes only during the middle stage, and 61 genes only during the late stage. Sixty-six (66) genes are differentially expressed in early and middle stages, 28 genes in early and late stages and 47 genes in middle and late stages. Sixty (60) genes are differentially expressed in all embryo development stages Click here for file

Additional file 6 **Differential expression of stage-specific genes during each time point of embryo development (from 2 to 22 DAP)**. To characterize the transcriptional changes taking place between developmental stages, a Volcano Plot with Student's t-test analysis was used to identify genes that were specifically expressed at only one time point when compared to UO (2 DAP vs UO, 4 DAP vs UO, etc) during embryogenesis from 0 to 22 DAP, with a p value ≤ 0.01 and a ≥ ± 2 fold cut-off. Click here for file
