# Supplementary material for: Transcription profiling of fertilization and early seed development events in a solanaceous species using a 7.7 K cDNA microarray from Solanum chacoense ovules
Source: BMC Plant Biol. 2010 Aug 12;10:174. doi: 10.1186/1471-2229-10-174 (PMC3095305; doi:10.1186/1471-2229-10-174)
Supplement: Additional file 1 — Quantitative PCR primers. Primer sequences used in the real time quantitative PCR expression analysis of selected genes representing candidates from the early, middle, and late stages of embryogenesis shown in figure 8. [file 1471-2229-10-174-S1.DOC]

**Supplemental Table 1**: Primer sequences used in the real time quantitative PCR expression analysis of selected genes representing candidates from the early, middle and late embryogenesis stages shown in figure 9.

| Genebank | Primer name | Primer sequence |
| --- | --- | --- |
| DN980725 | SV6_28H11-F | TTGTTGTGGTTCCAAGGTGA |
| DN980725 | SV6_28H11-R | GTAGGCCCTAGTGCTGTTGC |
| DN981910 | SV6_42C06-F | TCACCGTTGGCGTCTACATA |
| DN981910 | SV6_42C06-R | ACACCCAGCTTTCTCCAAT |
| DN978427 | SV5_50G09-F | TTGGAAGCCATGAGGAACT |
| DN978427 | SV5_50G09-R | GAAGGCGTGAGCTATGGAA |
| DN983138 | SV6_06F09-F | GCCAAGGTGTGTCCAAGAAT |
| DN983138 | SV6_06F09-R | GGTCAGACTCCCCTTCACAA |
| DN976716 | SV5_34H08-F | AGCAGCAGCAACAACAGAGA |
| DN976716 | SV5_34H08-R | AGCAAGGGCAACTTTGCTTA |
| DN983239 | SV6_07H10-F | CAGTGCCGAGTTGCAGATAA |
| DN983239 | SV6_07H10-R | TGTAAACGCTGTCGTTCTGG |
| DN978469 | SV5_51C03-F | GACATGGAACACACACTGC |
| DN978469 | SV5_51C03-R | CAAAATGCCTTCCACCAAG |
| DN979177 | SV5_58F03-F | GCCGGAGTTCAAGCTGTTA |
| DN979177 | SV5_58F03-R | CAAGTGGCGACTCTGTTTCA |
| DN976898 | SV5_36G10-F | AACGTCGTGACTGGGAAAAC |
| DN976898 | SV5_36G10-R | CGATTCGGCCTATTGGTTA |
| DN977330 | UBQ-F | GCTGGCAAGCAGTTGGAAGAT |
| DN977330 | UBQ-R | TGGATGTTGTAGTCCGCCAGA |
